# The Freshwater Sponge *Ephydatia fluviatilis* Harbours Diverse *Pseudomonas* Species (*Gammaproteobacteria*, *Pseudomonadales*) with Broad-Spectrum Antimicrobial Activity

**DOI:** 10.1371/journal.pone.0088429

**Published:** 2014-02-12

**Authors:** Tina Keller-Costa, Alexandre Jousset, Leo van Overbeek, Jan Dirk van Elsas, Rodrigo Costa

**Affiliations:** 1 Centre of Marine Sciences (CCMAR), University of Algarve, Faro, Algarve, Portugal; 2 Department of Ecology and Biodiversity, Utrecht University, Utrecht, The Netherlands; 3 Plant Research International, Wageningen University and Research Centre, Wageningen, The Netherlands; 4 Department of Microbial Ecology, Centre for Ecological and Evolutionary Studies, University of Groningen, Groningen, The Netherlands; 5 Microbial Ecology and Evolution Research Group, Centre of Marine Sciences (CCMAR), University of Algarve, Faro, Algarve, Portugal; Missouri University of Science and Technology, United States of America

## Abstract

Bacteria are believed to play an important role in the fitness and biochemistry of sponges (Porifera). *Pseudomonas* species (*Gammaproteobacteria*, *Pseudomonadales*) are capable of colonizing a broad range of eukaryotic hosts, but knowledge of their diversity and function in freshwater invertebrates is rudimentary. We assessed the diversity, structure and antimicrobial activities of *Pseudomonas* spp. in the freshwater sponge *Ephydatia fluviatilis*. Polymerase Chain Reaction – Denaturing Gradient Gel Electrophoresis (PCR-DGGE) fingerprints of the global regulator gene *gacA* revealed distinct structures between sponge-associated and free-living *Pseudomonas* communities, unveiling previously unsuspected diversity of these assemblages in freshwater. Community structures varied across *E. fluviatilis* specimens, yet specific *gacA* phylotypes could be detected by PCR-DGGE in almost all sponge individuals sampled over two consecutive years. By means of whole-genome fingerprinting, 39 distinct genotypes were found within 90 fluorescent *Pseudomonas* isolates retrieved from *E. fluviatilis*. High frequency of *in vitro* antibacterial (49%), antiprotozoan (35%) and anti-oomycetal (32%) activities was found among these isolates, contrasting less-pronounced basidiomycetal (17%) and ascomycetal (8%) antagonism. Culture extracts of highly predation-resistant isolates rapidly caused complete immobility or lysis of cells of the protozoan *Colpoda steinii*. Isolates tentatively identified as *P. jessenii*, *P. protegens* and *P. oryzihabitans* showed conspicuous inhibitory traits and correspondence with dominant sponge-associated phylotypes registered by cultivation-independent analysis. Our findings suggest that *E. fluviatilis* hosts both transient and persistent *Pseudomonas* symbionts displaying antimicrobial activities of potential ecological and biotechnological value.

## Introduction

Sponges (Porifera) are sessile filter-feeding organisms that primarily lack evasive or behavioural defence strategies [1]. Besides mechanical deterrence enabled by their spicules [2], sponges seem to mainly rely on chemical defence to prevent predation (*e.g.* by fishes and molluscs), avoid microbial biofilm formation and impede fouling [2]–[5]. There is increasing evidence that bacterial symbionts are the actual producers of many sponge-derived antagonistic metabolites [6]–[10], and this aspect has triggered much research interest in the diversity and bioactive potential of bacteria from marine sponges [9], [11], [12]. Conversely, knowledge of microbial communities in freshwater sponges remains limited. Their ubiquity in continental water bodies [13], coupled to recent molecular findings on highly selected communities and specific lineages of bacteria that inhabit them [14] make freshwater sponges valuable models in symbiosis research. Although inland water sponges likely synthesize less secondary metabolites than marine species [15], they are prolific producers of fatty acids, lipids and sterols. Indeed, more than 100 distinct such compounds have been recorded for freshwater sponges and some might be of bacterial origin [16]. Commensal bacterial communities may therefore fulfil important services required for the survival of their freshwater sponge host.

*Pseudomonas* species (*Gammaproteobacteria*, *Pseudomonadales*) are one ubiquitous group of metabolically versatile, eukaryote-associated bacteria with important effects on host health and survival, where they display a multitude of behaviours [17]. Often commensalistic, pseudomonads may act as opportunistic pathogens *e.g.* in plants [18], [19], fish [20], [21] and humans [22], [23]. In contrast, they are found in synergistic association with arbuscular mycorrhizae [24] and plant roots where they play beneficial roles in plant growth promotion and disease control [25], [26]. The two-component regulatory system GacS/GacA mediates the interaction between *Pseudomonas* spp. and their hosts. It controls the biosynthesis of several secondary metabolites and exoenzymes at the post-transcriptional level [25], [27]. Mutations in *gacA* and *gac*S genes induce phenotypic variation in *Pseudomonas* spp. [28], [29], affecting host colonization and persistence traits such as motility, biofilm formation, biosurfactant synthesis and protein secretion [28]–[31]. Previous studies demonstrated that the *gacA* gene is a high-resolution phylogenetic marker to the study of *Pseudomonas* spp. [32], [33].

Pseudomonads co-dominate the culturable fraction of the freshwater sponge microbiome [34] and have been previously detected in *E. fluviatilis* by cultivation-independent means [14]. Recently, Lipko et al. [35] reported on polyketide synthase (PKS)-encoding genes from a freshwater sponge pseudomonad. It is well known that *Pseudomonas* genomes are equipped with a wide range of secondary metabolite biosynthetic gene clusters, including PKS clusters [36], [37]. However, complete genome sequences from - and dedicated studies of - freshwater *Pseudomonas* spp. are scarce and our understanding of their diversity, secondary metabolite production capacity and adaptive strategies limited. Here, we combine culture-dependent and -independent methods to unveil the structure, diversity, and antimicrobial properties of *Pseudomonas* spp. in the freshwater sponge *Ephydatia fluviatilis*. To this end, PCR-DGGE fingerprinting of *Pseudomonas*-specific 16S rRNA and *gacA* genes was used to test the hypotheses of selectivity and temporal stability of *Pseudomonas* assemblages in the animal host. We further identify *Pseudomonas* species cultured from the sponge and determine their genome-wide diversity, antagonism towards several microorganisms and distribution/dominance across *E. fluviatilis* individuals. We finally address the potential biotechnological value of *E. fluviatilis* as a promising source of novel pseudomonads presenting antimicrobial activities.

## Materials and Methods

### Sampling

*Ephydatia fluviatilis* specimens were collected in the Vinkeveense Plassen lake (VP, (52°14′N, 4°57′E), an artificial lake located in the northwest of the province of Utrecht in the Netherlands on June 6, 2007 and June 4, 2008. The specimens were found at a depth of 9 m along a ∼60 m transect on woody material of a shipwreck scuttled at the diving point (‘zandeiland 4’) of VP and/or on zebra mussels (*Dreissenia polymorpha*) attached to wrecks and hard substrate. Each year, four sponge individuals (2007: SA – SD; 2008: SE – SH, about 10 g wet weight each) were sampled by scuba diving and placed in 50 mL falcon tubes filled with lake water. Four bulk water samples (W1–W4) were collected in sterile 500 mL flasks in year 2007 at about 3 m depth. This sampling scheme enabled us to address the hypothesis of selective shaping of *Pseudomonas* assemblages in *E. fluviatilis* (sponge *vs.* water comparison, 2007 samples) and to detect *Pseudomonas* phylotypes consistently associated with *E. fluviatilis* through time, if any (2007 *vs.* 2008 comparison). Further results on sponge *vs.* water comparisons for samples collected in 2008 have been described elsewhere [14]. Samples were transported to the laboratory (∼2.5 h) in a cooling box and immediately processed for DNA extraction and culturing. Because sampling involved invertebrate animals not representing endangered or protected species and did not occur within privately owned or protected areas, no specific permits were required for the described field studies. Sampling procedures were minimally intrusive and preserved sponge individuals at the field site.

### Total community DNA extraction

Total community DNA extraction from sponge and bulk water samples took place as described before [14]. Briefly, homogenates were obtained by grinding sponge samples with mortar and pestle. These were subjected to differential centrifugation to yield sponge-derived microbial cell pellets, which were used for DNA extraction with the Fast DNA® Spin Kit for Soil (Bio101, Q-Biogene, Heidelberg, Germany). The same kit was applied to extract DNA from water samples concentrated on a sterile 0.2 µm nitrocellulose membrane filter (Carl Roth GmbH, Karlsruhe, Germany).

### *Pseudomonas*-specific PCR-DGGE fingerprinting

Cultivation-independent analysis of *Pseudomonas* diversity in *E. fluviatilis* and bulk water DNA samples was carried out using PCR-DGGE primer systems and cycling conditions as previously described for the amplification of *Pseudomonas*-specific 16S rRNA [38] and *gacA* [33] gene fragments. All PCRs were performed on a Veriti^tm^ thermal cycler (Applied Biosystems, Foster City, CA). Denaturing gradient gel electrophoresis (DGGE) was performed with a PhorU-2 gradient system (Ingeny International, Goes, The Netherlands). *Pseudomonas*-specific 16S rRNA and *gacA* gene amplicons were applied onto polyacrylamide gels containing a 46.5–65% gradient of denaturants (100% denaturants defined as 7M urea and 40% formamide) and a 6–9% gradient of acrylamide [14]. Mixtures of 16S rRNA PCR products from five bacterial species (*Arthrobacter* sp., *Burkholderia* sp., *Enterobacter* sp., *Listeria* sp. and *Rhizobium* sp.) were applied at the edges of the 16S rRNA gel and used as standard to control run quality. The PCR-DGGE electrophoretic mobility of *gacA* gene fragments amplified from 36 *E. fluviatilis* derived *Pseudomonas* isolates shown to differ in whole genome content (see “BOX-PCR fingerprinting” below) was determined. These PCR products were pooled and used as a reference on the *gacA* gel to look for band matches in the sponge metagenomic DNA samples that could be assigned to these isolates. Electrophoresis was performed in 1x TAE buffer (pH 8.0) at 58°C and 140 V for 16 h. Gels were silver-stained [39] and air-dried at room temperature (RT). Processing of the gel images and multivariate statistical analysis of PCR-DGGE data were accomplished following the procedures of Costa et al. [40]. Briefly, the software package GelCompar 4.5 was used to calculate a “samples *vs.* band abundance” contingency table for each gel, which was then used as input data for multivariate statistics with Canoco for windows 4.5. Firstly, detrending correspondence analysis was applied to both 16S rRNA and *gacA* gene datasets to check the length of gradient in PCR-DGGE fingerprint variation. The linear model of distribution fitted both data best and, therefore, redundancy analysis (RDA) was chosen to estimate the extent to which the variation in the relative abundance of *Pseudomonas* DGGE bands are explained by the independent variables, namely sample origin and year of sampling.

### Isolation of fluorescent *Pseudomonas* spp. from *E. fluviatilis*

One gram of each sponge specimen sampled in year 2008 (SE – SH) was cut and homogenized in 4 mL sterile 0.85% saline solution using mortar and pestle. Serial dilutions were prepared and spread onto Gould's S1 (GS1) agar [41] plates in triplicate. Volumes of 1, 10 and 50 mL freshwater were filtered in triplicate per sample through sterile 0.2 µm nitrocellulose filters (47 mm; Carl Roth GmbH, Germany). Filters were then placed onto GS1 agar plates. All plates were incubated at 28°C. Fluorescent colony forming units (CFU) were counted under UV light after 2 and 5 days of incubation. Ninety colonies from sponge samples (18 to 26 per specimen) were randomly picked and purified with successive streaks on modified King's B (KB) agar plates (1.5% agar, 1.0% peptone, 0.75% glycerol, 0.075% K_2_HPO_4_, 0.075% MgSO_4_). Purified isolates were stored in KB broth with 20% glycerol at −80°C.

### Whole-genome diversity of *Pseudomonas* isolates

Two millilitre aliquots of cultures shaken overnight were centrifuged at 16,000 *g* for 20 min. The supernatant was discarded and genomic DNA extracted from the resulting cell pellets with the Wizard® Genomic DNA Purification Kit (Promega, Madison, WI, USA). To determine genotypic diversity within *Pseudomonas* isolates, whole-genome BOX-PCR genotyping was performed with the BOXA1R primer (CTA CGG CAA GGC GAC GCT GAC G) as previously described [42]. This primer is complementary to the boxA subunit of the conserved BOX element, a repetitive DNA sequence present in most gram-negative bacteria, including pseudomonads. Its selective amplification leads to a genotypic fingerprint that permits differentiation to the strain level. BOX-PCR amplicons were resolved by electrophoresis at 80 V on 2% agarose gels. Gels were visualized under UV light (256 nm) after 30 min staining in ethidium bromide (1 µg mL^−1^). Cluster analysis of the resulting BOX-PCR profiles was performed using the GelCompar II software (Applied Maths, Ghent, Belgium) as described elsewhere [42]. The Shannon measure of diversity (*H'*) was applied to estimate genotypic diversity within fluorescent pseudomonads isolated from each *E. fluviatilis* specimen.

### Identification and phylogeny of *Pseudomonas* isolates

Isolates displaying singular BOX-PCR profiles (singletons) and representatives of each BOX-PCR genotypic group determined by cluster analysis were selected for 16S rRNA and *gacA* gene sequencing. 16S rRNA gene fragments (∼1500 bp) were amplified with primers F8 (5′-AGA GTT TGA TCM TGG CTC AG-5′) and R1492 (5′-GGT TAC CTT GTT ACG ACT T-3′) [43] with 56°C annealing temperature in 30 PCR cycles. *GacA* gene fragments (575 bp) were amplified using the forward primer *gacA*-1F [33] and the reverse primer *gacA*-2 [32] as explained by Costa et al. [33]. 16S rRNA and *gacA* gene PCR products were sequenced at MACROGEN Inc. (Seoul, South Korea) using standard Sanger sequencing procedures. Sequences were edited with the Sequence Scanner Software version 1.0 (Applied Biosystems, Foster, CA, USA). Closest matches to sequence queries were identified using the blast algorithm of NCBI (http://blast.ncbi.nlm.nih.gov/Blast.cgi). Closest type-strain 16S rRNA gene relatives to each sequenced isolate were determined using the RDP II sequence match tool (http://rdp.cme.msu.edu/seqmatch) and served as reference for the tentative identification of the isolates at the species level. For single gene-based diversity assessments and phylogeny inference, 16S rRNA gene partial sequences were aligned using the SINA web aligner tool and imported into the ARB software. Analysis of *gacA* gene sequences succeeded through the construction of a *gacA* gene database in ARB with subsequent slow and accurate alignment using ClustalX. 16S rRNA and *gacA* gene alignments were manually corrected using the ARB sequence editor window. Phylip format alignments were used as input files for the generation of 16S rRNA and *gacA* gene sequence similarity matrices with the dnadist software, applying the Kimura-2 parameter as distance algorithm. Sequences were assigned operational taxonomic units (OTU) using DOTUR [44]. The frequency data assigned to a ‘unique’ OTU at 99%, 97% and 95% similarity levels were used to yield rarefaction curves for each gene. Maximum likelihood phylogenetic inference was carried out for the *gacA* gene dataset with the general-time reversible (GTR) evolutionary model with a discrete gamma-distribution of among-site rate variation (Γ4) and a proportion of invariant sites (I). This was regarded the best-fit model of nucleotide substitution for the dataset as determined by analysis run on MrModelTest version 2.3 [45]. Phylogenetic analysis and tree construction were conducted with the software package MEGA version five [46]. Sequences were deposited in the European Molecular Biology Laboratory database under the accession numbers HE794891–HE794933 (16S rRNA gene) and HE794934–HE794974 (*gacA* gene).

### Biofilm formation in static microcosms

An air-liquid (A-L)-interface assay for biofilm formation was performed using the static microcosm approach developed by Ude et al. [47]. Briefly, static microcosms consisted of glass tubes containing 5 mL of liquid KB. Fifty microliter inocula from overnight shaking cultures were used to start the biofilm formation assay. Tubes were incubated for 6 days, vibration-free, at 23°C with lids loosely attached to allow air exchange. Biofilm material, if present, was taken from the A-L interface with a wire loop and used to inoculate a second, fresh microcosm. If no biofilm was observed by naked eye or as material present on the wire loop, then the microcosm was vortex-shaken for 30 sec and a 50 µL aliquot was taken for inoculation of a new microcosm. All 90 isolates were tested in triplicate through three successive passages over a maximum of 18 days. A microcosm was scored as biofilm-positive if - at least in one replicate - material at the surface could be observed by eye (score +), or detected using a wire loop (score +/−). *Pseudomonas protegens* strain CHA0, which yields a mucilaginous biofilm under the tested circumstances, was employed as a positive control in the assays.

### *In vitro* antimicrobial activity

*In vitro* antagonistic activity of *Pseudomonas* spp. isolated from *E. fluviatilis* was tested towards the phytopathogenic fungi *Rhizoctonia solani* strain AG3 (basidiomycete) and *Fusarium moniliforme* strain CBS 218.76 (ascomycete), the phytopathogenic oomycete *Pythium ultimum* strain 67-1 and the commensalistic bacterium *Bacillus subtilis* F6 Rp^r^. For both fungi and *P. ultimum*, tests were performed in dual-culture assays as described before [48] on modified Potato Dextrose Agar (1.95% PDA, 0.5% Peptone, 1.5% Agar). Antagonistic potential against *B. subtilis* F6 Rp^r^ was tested following the agar plug assay procedure of Hentschel et al. [49]. All experiments were set up in triplicate and the degree of antagonism was estimated by measuring the size of the haloes of inhibition of *B. subtilis* after 2 days, of *P. ultimum* and *R. solani* after 4 days and of *F. moniliforme* after 7 days of incubation at 28°C. The growth of the target organisms in the absence of potential antagonists was monitored and used as blank. The soil-derived, root-colonizing bio-control strain *Pseudomonas protegens* CHA0, which inhibits all tested strains *in vitro*, was used as a positive control for antagonism in all trials and replicates.

### Resistance against protozoan predation

The resistance of *Pseudomonas* strains to predation by *Colpoda steinii* strain Sp1 was evaluated in a microtiter plate assay adapted from Jousset et al. [50]. *C. steinii* culture stocks were maintained in LB broth diluted 1∶10 in sterile amoeba saline (AS) solution [51] and purified in two centrifugation steps (1000 *g*, 1 min) prior to the start of the experiments. Fresh *Pseudomonas* cultures were incubated overnight with gentle agitation (50 rpm) at RT in 96-well plates containing 150 µL LB broth 1∶20 diluted in AS. Twenty microliters of overnight cultures were transferred to a new 96-well microtiter plate, each well containing 80 µL AS mixed with 20 µL of washed and active *C. steinii* suspension (set at final density of 1,000 cells mL^−1^). Plates were incubated with gentle agitation (50 rpm) at RT, and bacterial densities (OD_600_) in the presence and absence of *C. steinii* were measured at regular intervals over a 50 h period with an M200 plate photometer (Tecan, Männedorf, Switzerland). Bacterial sensitivity to predation was defined as the relative reduction of the OD_600_ value after its stabilization, usually at 22 h, in the presence of the protozoan compared to the initial OD_600_ value at 0 h readily after protozoan inoculation. Wells containing only bacterial isolates or protozoan cells were monitored in parallel to control for vitality and growth of the tested organisms. Bacteria with less than 40% reduction of the initial optical density after 22 h of exposure to *C. steinii* were counted as “predation-resistant”. The objective of this assay was to perform one single screening for resistant isolates to be used in the short-term toxicity assay against *C. steinii* (see below).

### Short-term toxicity of *Pseudomonas* spp. extracts toward *Colpoda steinii*

Predation-resistant isolates, determined as described above, were grown for 2.5 days in 20 mL LB broth (10x diluted in AS) at 27°C under shaking at 200 rpm. The cell suspensions were then transferred to 50 mL Falcon tubes containing 2.2 mL of 1 M HCl and vortexed, after which 20 mL ethyl acetate was added. Tubes were then shaken for 90 min at 37°C and centrifuged (1800 *g*, 10 min). The solvent-phase supernatants (∼10 mL) were carefully transferred to new tubes and dried to air. The dried bacterial extracts were resuspended in 100 µL methanol and kept at −20°C. One microliter volumes of the respective cell extracts were added to 100 µL freshly grown *C. steinii* suspensions in 96-well microtiter plates, and suspensions were gently homogenized by shaking. Controls were monitored in parallel, replacing the cell extracts by 1 µL methanol. The extract of *P. protegens* CHA0 grown under the same conditions was included as positive control. The effect of extracts (and methanol) on *C. steinii* was evaluated in duplicate after 5, 15, 30, 60 and 120 min, respectively, using an inversion microscope (Axiovert 25, Carl Zeiss, Jena, Germany; magnification ×200 and ×400).

### Multivariate analysis of antagonistic properties

Multivariate analysis was performed with the objective of grouping the *Pseudomonas* isolates based on their profiles of antagonistic features. These were determined, per isolate, as the collective scores obtained in antibacterial, antifungal, anti-oomycetal and predation resistance in *in vitro* assays (semi-quantitative data), along with biofilm formation tests as described above, in accordance with the approaches of Costa et al. [33] and Adesina et al. [52]. Thus, the 90 *Pseudomonas* isolates were introduced in the statistics as samples, whereas their respective antimicrobial properties served as descriptors of each sample (dependent variables). The affiliation of *Pseudomonas* isolates to a certain *E. fluviatilis* specimen (i.e. specimens E, F, G, H) was used as a nominal (i.e. binary), independent variable. This way, correlations between antimicrobial properties and their association with each *E. fluviatilis* specimen could be explored. Analyses were carried out using the software package Canoco for Windows 4.5. After preliminary inspection of overall dataset variation by detrended correspondence analysis (DCA), principal components analysis (PCA) was selected as the most appropriate ordination method (linear, unconstrained) to be used in the analysis [53]. PCA was run with focus on inter-sample distances and results were illustrated in an ordination diagram.

## Results

### Cultivation-independent analysis of *Pseudomonas* communities

**Figure 1:**
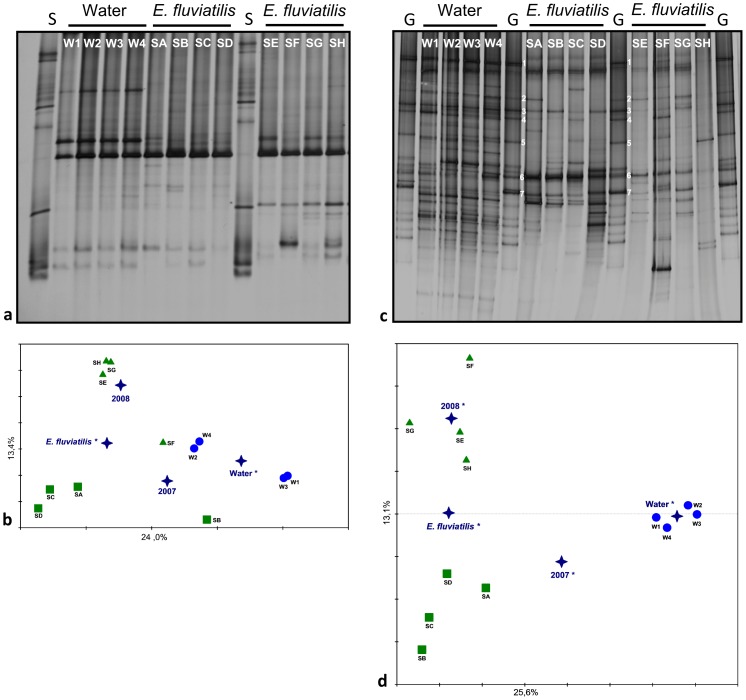
PCR-DGGE fingerprints. *Pseudomonas* 16S rRNA (a) and *gacA* (c) gene fragments amplified from *Ephydatia fluviatilis* (2007: SA-SD and 2008: SE-SH) and lake water (2007: W1–W4) and their respective ordination diagrams generated by redundancy analysis (b, d) are shown. 16S rRNA (S) and *gacA* (G) gene standards were applied on the corresponding gels to control the DGGE run (see methods for standard compositions). Numbers 1 to 7 show some of the PCR-DGGE *gacA* gene bands amplified from *E. fluviatilis* metagenomic DNA (cultivation-independent approach) that matched *gacA* gene electrophoretic mobilities from *E. fluviatilis*-derived *Pseudomonas* isolates (cultivation-dependent approach). In ordination diagrams, green squares and triangles correspond to *E. fluviatilis* samples from 2007 and 2008, respectively; and blue circles to lake water samples. Blue stars: centroid positions of the independent variables *E. Fluviatilis*, lake water and year of sampling. Independent variables found to significantly influence PCR-DGGE band profiling variation are marked with an asterisk.

PCR-DGGE analysis of *Pseudomonas* assemblages using specific 16S rRNA gene primers revealed low levels of band richness in all samples (Fig. 1a). Two up to three dominant bands in bulk water fingerprints and a single dominant band in *E. fluviatilis* profiles were visible, with fingerprints in general displaying less than 10 detectable bands. All profiles were characterized by low and statistically similar (*p* = 0.827; One-Way-ANOVA) Shannon diversity indices, with values of 1.673±0.35, 1.543±0.26 and 1.562±0.35 (means ± SD, *N* = 4) obtained for bulk water (2007) and *E. fluviatilis* (2007, 2008) samples, respectively. Regarding community structure as revealed by 16S rRNA gene fingerprinting, Monte Carlo permutation tests confirmed sample origin as a significant factor differentiating the profiles (*p* = 0.048 and *p* = 0.044 for the independent variables “water” and “*E. fluviatilis*”, respectively); however, yearly variation in sponge profiles was not significant (*p* = 0.132 and *p* = 0.142 for the independent variables “2007” and “2008”, respectively; Fig. 1b). In contrast with 16S rRNA gene analyses, the relative abundance of some *Pseudomonas gacA* bands from bulk water was enhanced in *E. fluviatilis* while several other bulk water *gacA* bands could not be readily detected in the sponge samples, resulting in reduced *gacA* band diversity in the latter PCR-DGGE fingerprints in comparison with the former (Fig. 1c). Significantly higher (*p*<0.001; *F*_2,9_ = 19.404; OW-ANOVA followed by Tukey-test) *gacA* diversity indices were found in bulk water (3.210±0.13) than in *E. fluviatilis* profiles from years 2007 (2.540±0.146) and 2008 (2.402±0.28). Diversity measures obtained for sponge *gacA* fingerprints from 2007 and 2008 were statistically similar. Monte Carlo permutation tests confirmed the independent variables “water” and “*E. fluviatilis*” to significantly differentiate the PCR-DGGE profiles (*p* = 0.002 and 0.006, respectively), with samples belonging to these microhabitats clearly separated along the horizontal axis of the ordination diagram (Fig. 1d). As opposed to 16S rRNA gene fingerprinting, year of sampling did contribute significantly to distinguish the *gacA* fingerprints of *E. fluviatilis* specimens (*p* = 0.03 for both independent variables 2007 and 2008, respectively). Overall, PCR-DGGE fingerprinting data showed statistically higher *Pseudomonas gacA* gene diversity than predicted by 16S rRNA gene analysis in both water (*p* = 0.003; *F*_1,3_ = 77.844; OW-Repeated Measures-ANOVA followed by Tukey-test) and *E. fluviatilis* (*p* = 0.002; Two-Way-RM-ANOVA followed by Tukey-test) samples.

### Isolation of fluorescent *Pseudomonas* spp. from *E. fluviatilis*

Numbers of fluorescent *Pseudomonas* were low in the VP bulk water samples, ranging from 3.1–4.1×10^0^ CFU mL^−1^. In contrast, numbers in *E. fluviatilis* specimens were in the range of 1.5–3.6×10^3^ CFU g (fresh wt)^−1^, surpassing counts obtained for bulk water by *c.* 3 orders of magnitude. In total, 90 isolates were obtained from *E. fluviatilis* specimens sampled in 2008 and thoroughly inspected for diversity, biofilm formation capacity and antimicrobial activities.

### Whole-genome diversity and identity of *Pseudomonas* isolates

**Figure 2:**
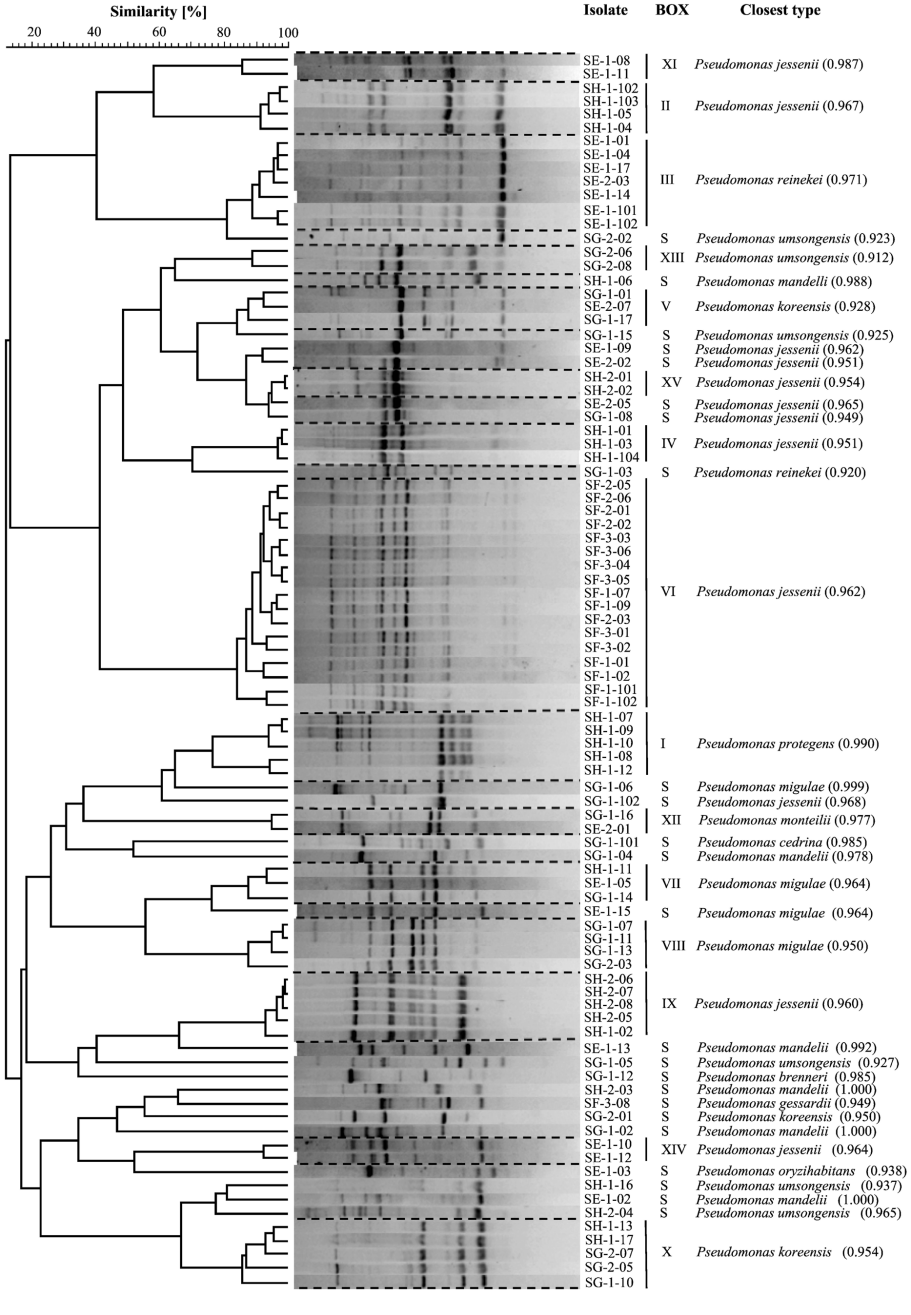
BOX-PCR genotyping of fluorescent *Pseudomonas* spp. isolated from *Ephydatia fluviatilis*. All 15 BOX-PCR clusters (BOX I–XV) and 24 singleton (S) profiles identified in this study are shown. Closest type strain 16S rRNA gene relatives (“Closest type”) present at the RDP database are given, with their sequence match scores (see Table S1 for definition and comparison with percent similarities) between brackets.

BOX-PCR genotyping (Fig. 2) revealed 15 fingerprint clusters (BOX I–XV) containing 2 to 17 isolates next to 24 fingerprints represented by one isolate (singletons), thus yielding 39 genotypes among the 90 surveyed isolates. Genotypic diversity of *Pseudomonas* isolates varied substantially across the source *E. fluviatilis* specimens. Eleven of the 15 BOX-PCR clusters were exclusive to a given sponge individual. The four remaining genotype clusters (BOX V, VII, X and XII) contained isolates retrieved from either 2 or 3 different sponge specimens (Fig. 2). Many BOX-PCR singletons (10 of 24) were detected in *E. fluviatilis* specimen SG. This sponge sample yielded the highest BOX-PCR-based Shannon diversity index (Table 1). Forty-three representative isolates covering the 39 observed BOX-PCR profiles were all identified as members of the genus *Pseudomonas* by 16S rRNA gene sequencing (Table S1). Twelve of the 39 BOX-PCR genotypes shared 100% 16S rRNA gene sequence similarity with type strains of species such as *P. mandelii*, *P. migulae*, *P. monteilii*, *P. reinekei*, *P. brenneri* and *P. cedrina* (Table S1). Overall, 40 and 13 isolates resembled *P. jessenii* and *P. umsongensis* based on >99% 16S rRNA gene similarities between genotype representatives and type strains of these species (Tables S1 and S2). These isolates encompassed several BOX-PCR genotypes and antagonistic profiles, representing the most prominent species tentatively identified in this study (Table S2). The four BOX-PCR genotypes retrieved from more than one sponge specimen had highest resemblance to *P. koreensis* (BOX V), *P. migulae* (BOX VII), *P. umsongensis* (BOX X) and *P. monteilii* (BOX XII) type strains (Fig. 2, Table S1). BOX cluster I comprised five isolates from specimen SH (Fig. 2). The biological control strains Pf-5 and CHA0 (T), belonging to the recently described species *P. protegens* (Ramette et al., 2011), exhibited the highest genetic similarity to this group (Table S1). Seventeen of the 18 isolates from sponge specimen F belonged to the same BOX-PCR cluster (VI). *P. jessenii* CIP 105274 was the closest type strain to this group (Table S1).

**Table 1:** Genotypic diversity, biofilm capacity and *in vitro* antagonistic activity of fluorescent *Pseudomonas* isolates from *Ephydatia fluviatilis*.

Specimen^1^	N^2^	H'^3^	Biofilm^4^	*R. sol.*	*P. ult.*	*F. mon.*	*B. sub.*	*C. stein.*
**SE**	21	2.264	11	6	11	1	9	5
**SF**	18	0.215	18	6	3	6	13	10
**SG**	25	2.754	12	2	7	0	11	5
**SH**	26	2.192	11	1	8	0	11	12
**All**	90	1.404	52	15	29	7	44	32

1 SE, SF, SG, SH – *Ephydatia fluviatilis* individuals collected in 2008.

2 N - total number of isolates per sponge sample.

3 H' - BOX-PCR-based (Fig. 2) genotypic diversity of fluorescent *Pseudomonas* per sponge sample, estimated with the Shannon diversity index.

4 All other values refer to the number of isolates from each sponge individual tested positive for biofilm formation (Biofilm), antagonism towards *Rhizoctonia solanii* AG3 (*R. sol.*), *Pythium ultimum* 67-1 (*P. ult.*), *Fusarium moniliforme* CBS 218.76 (*F. mon.*) and *Bacillus subtilis* F6 Rpr (*B. sub.*), and predation resistance towards *Colpoda steinii* Sp1 (*C. stein.*).

### Diversity and phylogeny of *Pseudomonas* isolates

**Figure 3:**
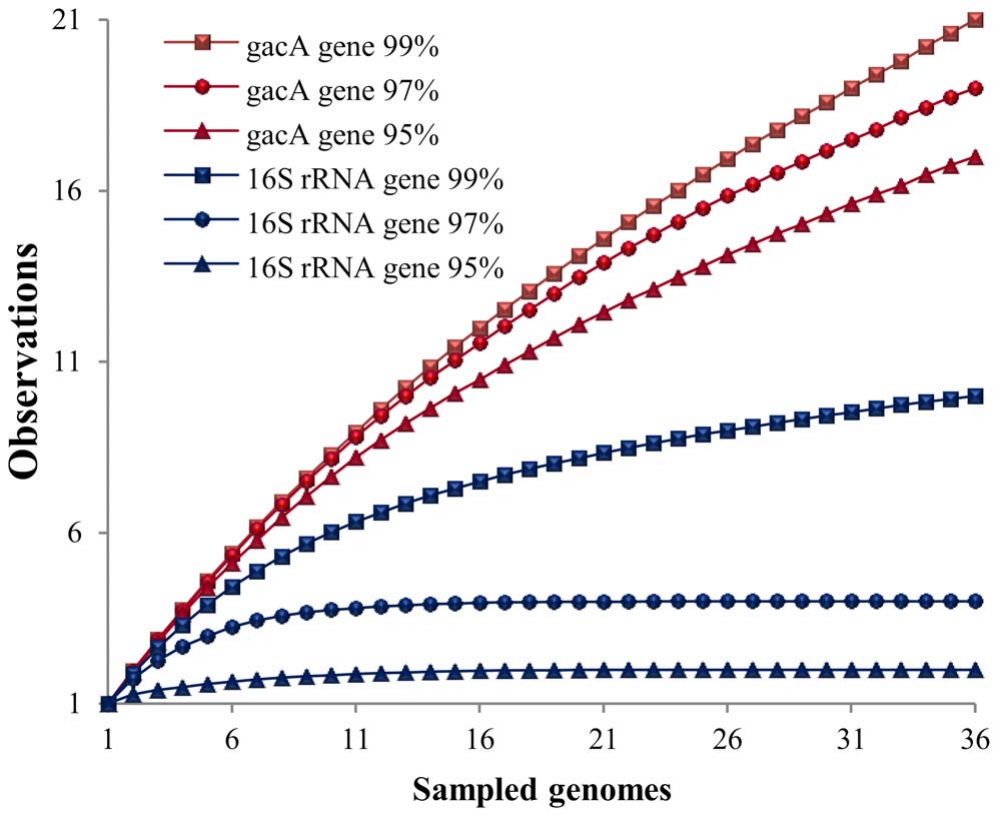
Rarefaction curves. Shown are operational taxonomic units (OTUs) observed at 99%, 97% and 95% similarity levels for partial 16S rRNA (blue) versus *gacA* (red) gene sequences of *E. fluviatilis*-derived, fluorescent *Pseudomonas* isolates encompassing 36 different genomes as determined by BOX-PCR fingerprinting (Fig. 2).

**Figure 4:**
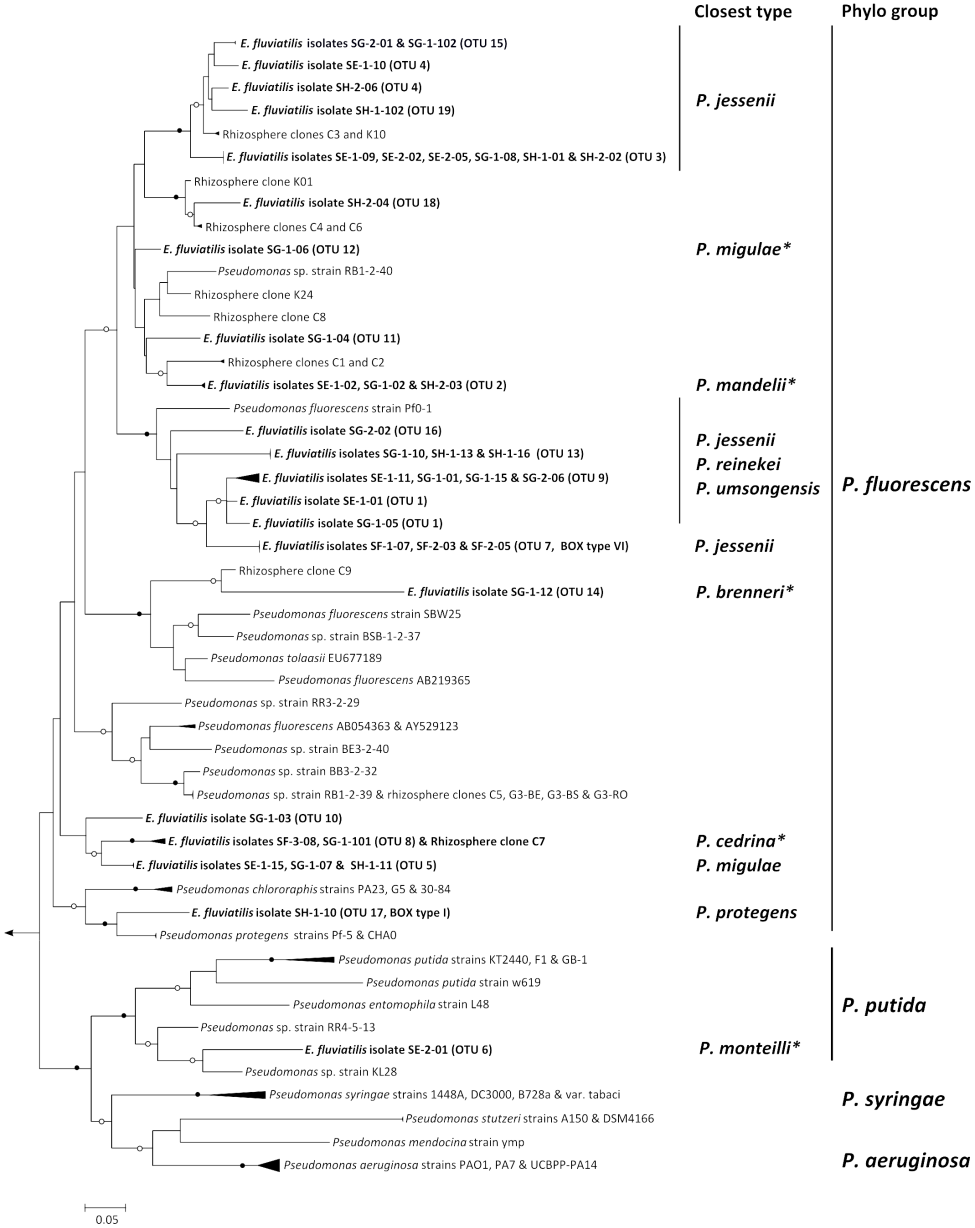
Maximum likelihood phylogenetic tree of *Pseudomonas gacA* genes. *GacA* gene sequences of *E. fluviatilis*-derived *Pseudomonas* isolates (bold) and of most (>90%) isolates and clones present in relevant databases are shown. In brackets after the isolates' entries are their corresponding *gacA* operational taxonomic units (OTU) as inferred by rarefaction analysis at 97% similarity threshold (Fig. 3). “Closest type” shows the 16S rRNA gene identity of the nearest type strain to relevant tree entries (see Table S1 for a complete list and details). An asterisk indicates 100% 16S rRNA gene similarity between isolates and closest type strains. “Phylo group” refers to major, super-specific *Pseudomonas* phylogenetic lineages as determined by Mulet et al. (2010). Open and close circles on tree nodes correspond to bootstrap values ≥70% and 90%, respectively. The scale bar indicates 5% nucleotide substitution per site. The tree was rooted with the *gacA* gene homologue of *Burkholderia pseudomallei* strain K96243.

Nucleotide heterogeneities among partial, high quality 16S rRNA and *gacA* gene sequences obtained for 36 *Pseudomonas* isolates were compared. These isolates represented all 15 BOX-PCR clusters and 21 of the 24 single BOX-PCR genotypes found in our survey (Fig. 2). A plot of observed operational taxonomic units (OTUs) *vs.* the number of compared sequences yielded dissimilar rarefaction curves for the 16S rRNA and *gacA* genes, regardless of the cut-off values used to define OTUs (Fig. 3). At 99% similarity, about twice the number of OTUs - 21 *vs.* 10 - was observed for the *gacA* gene sequences in comparison with the 16S rRNA gene sequences. This difference was more drastic when OTU numbers were compared at 97 and 95% similarity thresholds: only 4 and 2 OTUs were found for the 16S rRNA gene, whereas the *gacA* curve at these cut-off values yielded 19 and 17 OTUs, respectively (Fig. 3). *GacA* gene sequencing not only revealed higher diversity within the studied strains than inferred by the 16S rRNA gene-based method, with overall mean nucleotide *p*-distances of 15.4 and 2.1% registered for both genes, respectively, but also resulted in up to 18 novel, non-redundant *gacA* gene sequences added to public databases at a 97% gene similarity threshold (Fig. 4). Among them are the first *gacA* gene sequences assigned to species such as *P. migulae* (OTU 12), *P. mandelii* (OTU 2), *P. brenneri* (OTU 14), *P. monteilli* (OTU 6) and *P. cedrina* (OTU 8). Two well-supported phylogenetic clusters contained diverse and novel *gacA* genes that moderately resembled those of uncultured (clones C3 and K10, “*P. jessenii*” cluster) and cultured (*P. fluorescens* Pf0-1; “*P. jessenii*, P., *reinekei*, *P. umsongensis*” cluster) rhizosphere pseudomonads (Fig. 4). *GacA* gene phylogenetic inference was also helpful in discriminating isolate SH-1-10 (BOX I) from its closest relatives *P. protegens* Pf-5 and CHA0, and in depicting high nucleotide heterogeneity among several *P. jessenii*-like strains (Fig. 4), what could not be achieved by 16S rRNA gene analysis.

### Biofilm formation and *in vitro* antagonistic activities

**Figure 5:**
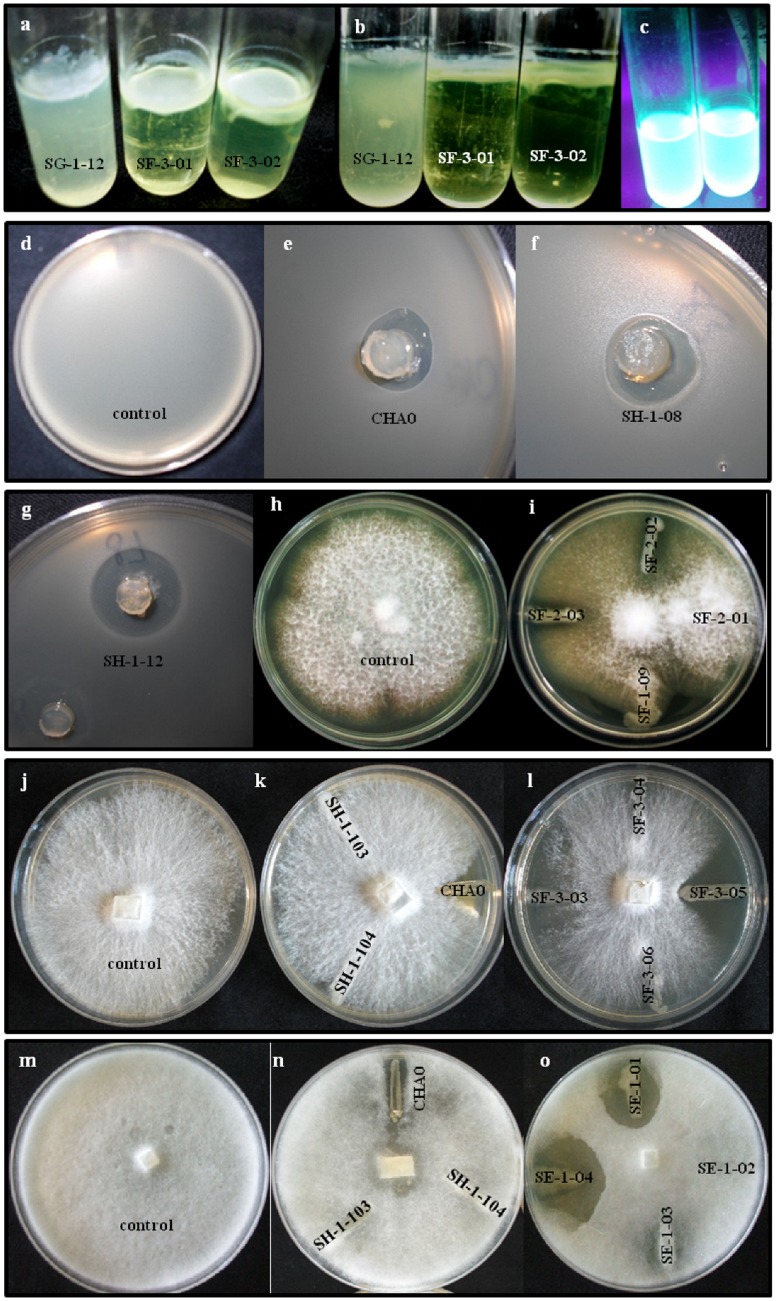
Biofilm formation and *in vitro* antifungal, anti-oomycetal and antibacterial activity of *Pseudomonas* spp. isolated from *Ephydatia fluviatilis*. Top-view (a) and lateral view (b) of a flocculent-type biofilm (SG-1-12) and mucilaginous-type biofilms (SF-3-01; SF-3-02) at the air-liquid interface in a test-tube; (c) example of two fluorescent *Pseudomonas* isolates under UV-light in liquid King's B medium. Inhibition of *Bacillus subtilis* (d–g), *Fusarium moniliforme* (h,i), *Rhizoctonia solani* (j–l) and *Pythium ultimum* (m–o) was assayed on PDA plates. *Pseudomonas protegens* strain CHA0 was used as a positive control in all tests (*e.g.*, e,k,n). To note is the inhibition of *R. solani* growth by *P. jessenii* isolates SF-3-03 and SF-3-05 (l) and of *P. ultimum* by *P. reinekei* isolates SE-1-01 and SE-1-04 (o). *P. protegens* isolates, here represented by isolates SH-1-08 (f) and SH-1-12 (g), displayed conspicuous antagonism towards *B. subtilis*.

Of the 90 isolates examined, 52 (58%) produced observable biofilms within 15 days of incubation. All isolates of BOX-PCR groups I (*P. protegens*-like) and VI (*P. jessenii*-like) evolved a detectable biofilm in the first microcosm after 6 days. The phenotypes of the produced biofilms could be categorized as either flocculent or mucilaginous (Fig. 5; Table S2). The latter type was found more frequently, in 40 (77%) of the 52 biofilm-producing isolates.

*In vitro* antagonistic activity against *R. solani*, *F. moniliforme*, *P. ultimum* or *B. subtilis* was observed for 68 of the 90 *Pseudomonas* isolates (75%), although none of them displayed antagonism towards all target organisms simultaneously (Table S2). The number of antagonists found was different for each test organism and sponge sample (Table 1). A higher proportion of isolates displayed antagonism against *P. ultimum* (32%) and *B. subtilis* (49%) than against the soil fungi *R. solani* (17%) and *F. moniliforme* (8%). Of the 7 isolates antagonistic to *F. moniliforme*, 6 belonged to BOX-PCR cluster VI (*P. jessenii*). Twenty-one isolates (23%) were strongly inhibitory towards one or more of the model targets (Table S2). Isolates with conspicuous antagonism were SE-2-03, SE-2-07 and SF-3-05 against *R. solani* and SE-2-07, SE-1-01, SE-1-04 and SG-2-01 against *P. ultimum* (Fig. 5, Table S2). Noteworthy was also the strong inhibition showed by all *P. protegens*-like isolates towards *B. subtilis* (Fig. 5; Table S2).

**Figure 6:**
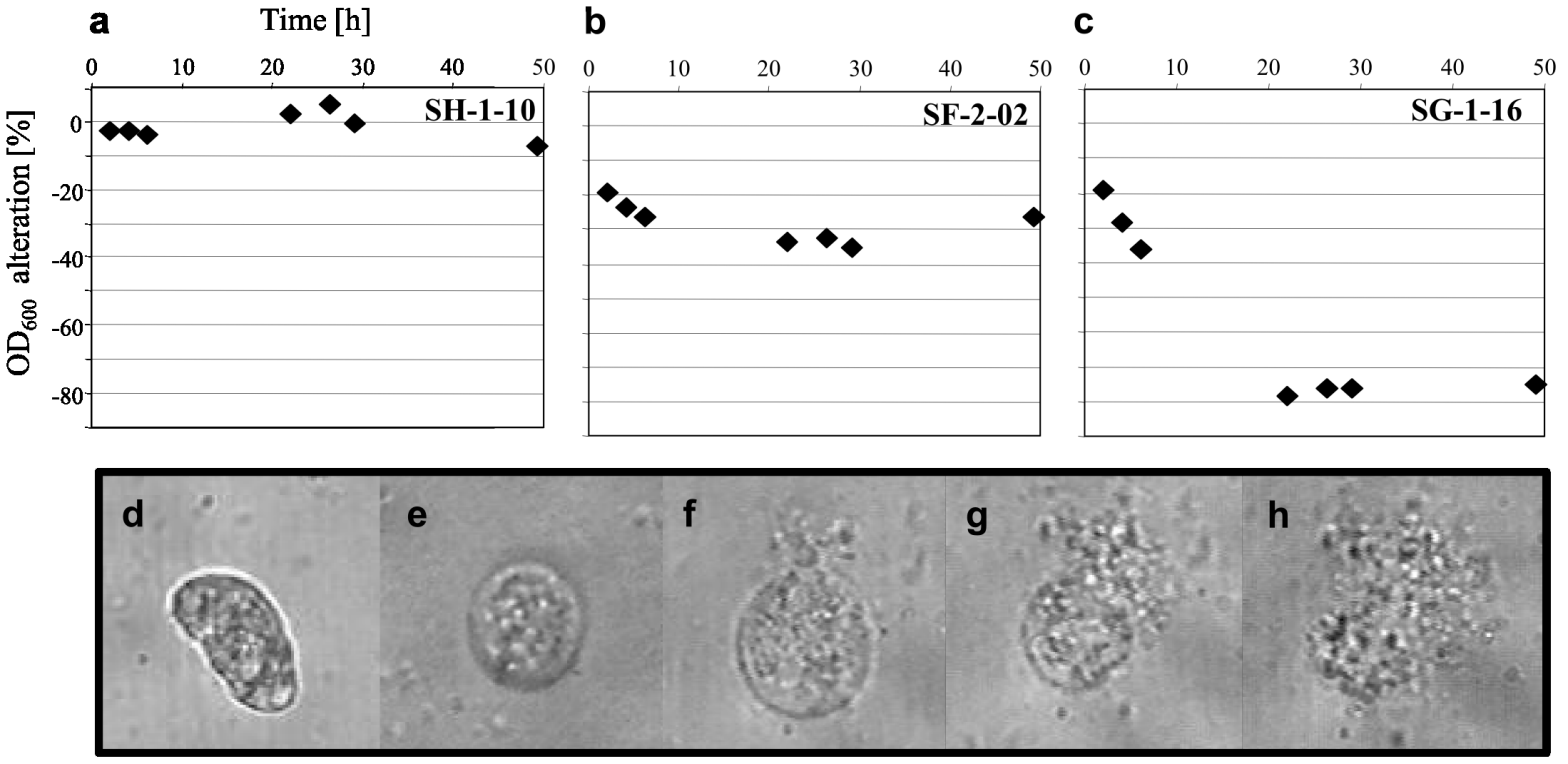
Predation resistance and toxicity to *Colpoda steinii* by *Pseudomonas* spp. isolated from *Ephydatia fluviatilis*. Predation resistance assays (a–c) determined the effect of the ciliate predator *Colpoda steinii* Sp1 on the growth of *Pseudomonas* cultures, estimated by measuring OD_600_ alteration over time. Representative isolates of three categories are shown: (a) no effect of *Colpoda steinii*, strong resistance of *P. protegens* isolate SH-1-10; (b) *C. steinii* causes slight depletion on a *Pseudomonas* culture: moderate resistance of *P. jessenii* isolate SF-2-02; (c) *C. steinii* depletes the *Pseudomonas* culture at the initial stage of the assay: no resistance exhibited by *P. monteilii* isolate SG-1-16. *Colpoda steinii* cells were then challenged with cell extracts from *Pseudomonas* isolates highly resistant to *C. steinii* predation (d–h). (d) active *C. steinii* cell; (e) deformed, immobile *C. steinii* cell; (f–h) cell lysis of *C. steinii*. Light microscopy magnification ×400.

When offered as a prey to the ciliate *C. steinii* Sp1, 35% of the isolates (32/90) resisted predation (Table 1, Fig. 6). *P. protegens*-like isolates (BOX I, Fig. 2), isolates SG-1-02 and SH-1-06 (*P. mandelli*) and isolates SH-1-17 (*P. umsongensis*) and SH-2-01 (*P. jessenii*, BOX XV) were highly efficient in withstanding protozoan predation, whereas *P. jessenii*-like isolates of BOX-PCR group VI showed variable results (Table S2).

**Figure 7:**
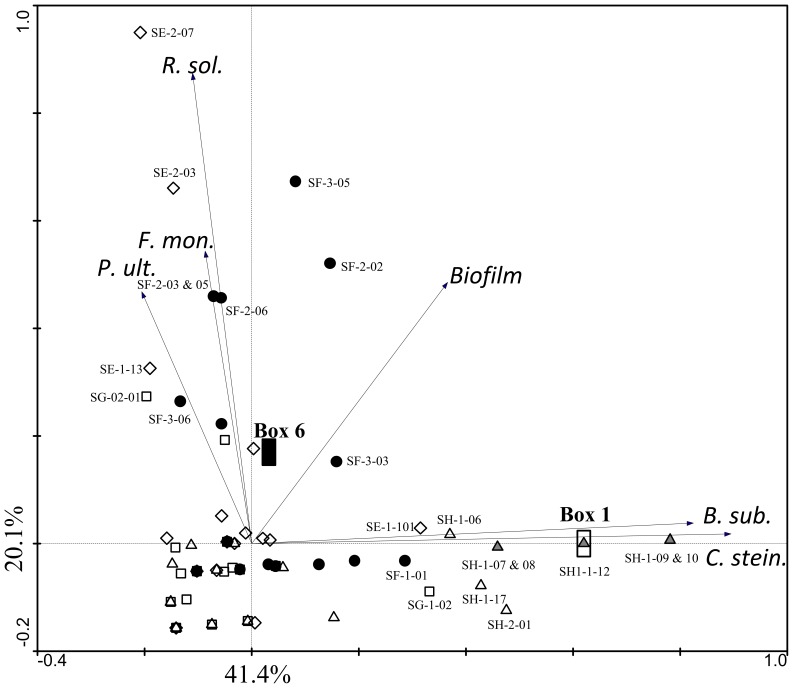
PCA ordination biplot of *Pseudomonas* isolates (symbols, n = 90) and their antimicrobial properties (arrows). Ranks of intensity (semi-quantitative data) were created to register the data on *in vitro* antagonistic activity towards *Rhizoctonia solani* (*R. sol.*), *Fusarium moniliforme* (*F.mon.*), *Pythium ultimum* (*P. ult.*), *Bacillus subtilis* (*B. sub.*), *Colpoda steinii* (*C. stein.*) and the ability of biofilm production in still microcosms (*Biofilm*). Arrows pointing in the same direction approximate properties that share positive correlation. Pairs of properties whose arrows diverge at ≥90° possess no or negative (linear) correlation. The differences in the frequencies of individual antimicrobial traits across the data are mirrored by the lengths of their arrows, with dominant traits (that is, scored more often and/or with higher intensity) displaying longer arrows than those with small score values. Isolates placed next to the tip of any given arrow possess a high score value for the corresponding antimicrobial trait. Empty diamonds: *Pseudomonas* isolates of sponge individual E. Solid circles: *Pseudomonas* isolates of sponge individual F. Empty squares: *Pseudomonas* isolates of sponge individual G. Triangles: *Pseudomonas* isolates of sponge individual H. Grey triangles highlight antagonists belonging to BOX-PCR group I (Box 1, *P. protegens*), found to display similar phenotypes. Isolates displaying strong antagonism have their identification codes placed next to their symbols. Centroid positions for isolates sharing the BOX-PCR types I (*P. protegens*) and VI (*P. jessenii*) are shown. Note the phenotypic variation within some of the isolates belonging to the latter category (black circles labelled with isolates' codes, see also Table S2).

To group the isolates in respect with their antimicrobial traits, a PCA ordination triplot was created (Fig. 7). The diagram privileges visualization of highly antagonistic isolates as opposed to those showing weak or moderate inhibitory scores. The weaker the antagonistic profile, the closer a given isolate is from the diagram's intercept. This way, the coincident and strong antagonism against *C. steinii* and *B. subtilis* exhibited by *P. protegens*-like isolates (BOX I) grouped them apart from most other strains. Five of the 7 isolates clearly antagonistic to *F. moniliforme* also inhibited the growth of *R. solani*, and this trend could be depicted by exploratory ordination analysis (Fig. 7). Strong *B. subtilis* and *C. steinii* antagonism had low correlation with pronounced antifungal and anti-oomycetal activities, resolving highly antagonistic *Pseudomonas* isolates in two distinct functional groups (Fig. 7). The arrow representing biofilm formation in the ordination diagram highlights the positive correlation between biofilm production capacity and the detection of antagonistic activity among the studied strains. Indeed, strains presenting no or rather weak antagonistic profiles, placed next to the diagram's intercept (Fig. 7), usually did not produce an observable biofilm (Table S2). No association between a given antagonistic attribute and the origin of the isolates (i.e. sponge specimens E, F, G, H) was found.

### Short-term responses of active *Colpoda steinii* to *Pseudomonas* spp. cell extracts

Active *Colpoda steinii* Sp1 cells were challenged with cell extracts from selected predation resistant *Pseudomonas* isolates (n = 20; Table S2). Cell extracts of *P. protegens* strain CHA0 (positive control) caused immobility of all *C. steinii* cells within 5 min and cell lysis within 120 min of observation. As negative and blank controls, *C. steinii* cultures – either pure or provided with 1 µL methanol, respectively - were monitored in parallel to each tested isolate. In most cases, methanol had no effect on the agility of *C. steinii* cells. For about 10% of the tested isolates, *C. steinii* cells with slower motility were observed in the first 30 min of investigation, but recovered within the given observation time (2 h). Conversely, *C. steinii* cells were rapidly affected by extracts of all *Pseudomonas protegens*-like isolates (Fig. 2, BOX I), *P. jessenii*-like isolates SF-2-02 and SF-3-03 (BOX VI), and isolates SG-1-02, SH-1-17 and SH-2-01 (Table S2). Within five to 15 min after provision of these extracts, all *C. steinii* cells became immotile, contrasting their observed motility in the controls. After 2 h of treatment, lysis (Fig. 6) was initiated in *C. steinii* cells provided with extracts of *P. protegens*-like isolates, and 24 h later not a single unbroken *C. steinii* cell could be recognized in these treatments. In certain cases (e.g. cell extracts of isolates SH-1-08 and SH-1-12), the slowing-down of the cilia-mediated movement was easily traced under the microscope. Interestingly, extracts of isolates SF-2-02, SH-1-17 and SH-2-01, all causing 100% immobility of *C. steinii* cells (Table S2), were not able to initiate lysis, and even 48 h after start of the experiment, the ciliate cells were intact.

### Linking cultivation-independent and -dependent analyses

We conducted PCR-DGGE *gacA* gene fingerprinting of an artificial, culturable *Pseudomonas* community composed of 36 of the 39 distinct BOX-PCR genotypes observed in Fig. 1. These 36 isolates presented high quality 16S rRNA and *gacA* gene sequences for comparative gene heterogeneity analyses (Fig. 3). PCR-DGGE profiling of their *gacA* genes revealed at least 16 clearly identifiable bands (see *gacA* gene standard - lanes “G” – in Fig. 1), approaching the number of OTUs determined by *gacA* gene sequence rarefaction analyses at 95 and 97% similarity thresholds for the same group of strains (Fig. 3). Several *gacA* PCR-DGGE bands obtained from pure cultures matched the electrophoretic mobility of bands detected in bulk water and *E. fluviatilis* metagenomic DNA profiles (Fig. 1). Seven of these bands are highlighted (Fig. 1), exemplifying different scenarios regarding (1) their frequency and dominance across water and sponge profiles, and (2) the antimicrobial features of *Pseudomonas* isolates matching their positions on DG-gels (Table 2). Interestingly, some of the isolates cultured from *E. fluviatilis* matched dominant bands from water profiles that could not be readily detected in the sponge fingerprints (e.g. bands 1 and 5, Fig. 1, Table 2). Further, PCR-DGGE bands consistently found in both water and *E. fluviatilis gacA* profiles (e.g. bands 2, 3, 4 and 6) corresponded to isolates identified as *P. oryzihabitans*, *P. jessenii* and *P. mandelii* that, collectively, displayed manifold antimicrobial activities (see Table 2 for details). Bands 6 and 7 are examples of *Pseudomonas* phylotypes enriched in *E. fluviatilis* in comparison with bulk water *gacA* profiles. The latter phylotype displayed an uneven pattern of occurrence across *E. fluviatilis* individuals and matched the *gacA* electrophoretic mobility of highly antagonistic and cytotoxic *P. protegens*-like strains (Fig. 2, BOX I; Table 2, Table S2).

**Table 2:** Matching PCR-DGGE *gacA* bands from total community and pure culture DNA samples.

Band^1^	PCR-DGGE feature	Matching Isolates	BOX^2^	OTU^3^	Phenotype	Closest type
1	Dominant in all water profiles; not detected in *E. fluviatilis* profiles	SG-1-10; SH-1-16	S; S	13	Biofilm formers, moderate antagonism and resistance against *B. subtilis* and *C. steinii*	*P. umsongensis*
2	Present in all water and several *E. fluviatilis* profiles	SE-1-03	S	n.a.	Antagonistic towards *B. subtilis* and resistant against *C. steinii*	*P. oryzihabitans*
3	Present in all water and *E. fluviatilis* profiles	SG-1-15; SG-2-06	S; XIII	9	Biofilm formers with no conspicuous antagonistic traits	*P. umsongensis*
4	Present in all water and several *E. fluviatilis* profiles	SF-1-07 and alike (n = 17)	VI	7	Biofilm formers antagonistic towards *F. moniliforme*, *R. solani* and *B. subtilis*	*P. jessenii*
5	Present in all water profiles; not detected in *E. fluviatilis*	SE-1-02; SG-1-02; SH-1-06	S; S; S	2	Resistant/cytotoxic to *C. steinii*; suppressive towards *P. ultimum*	*P. mandelii*
6	Detected in water and enriched in *E. fluviatilis*	SH-1-102; SH-1-103	II; II	19	Suppressive towards *P. ultimum*	*P. jessenii*
7	Not detected in water; enriched in some *E. fluviatilis* profiles	SH-1-10 and alike (n = 5)	I	17	Biofilm formers strongly antagonistic towards *B. subtilis* and resistant/cytotoxic to *C. steinii*	*P. protegens*

1 Band numbering as provided in Fig. 1c.

2 BOX-PCR grouping as determined in Fig. 2. S, singletons.

3 *GacA* gene operational taxonomic units as shown in Fig. 4.

n.a., not applicable.

## Discussion

This is the first dedicated study of *Pseudomonas* symbionts in freshwater sponges. Our first objective was to explore the diversity, selectivity and temporal stability of *Pseudomonas* spp. associated with *Ephydatia fluviatilis*. We reveal previously unsuspected complexity of *Pseudomonas* assemblages in a freshwater ecosystem by including an alternative phylogenetic marker – the *gacA* gene – in our cultivation-independent procedures. In contrast, the conventional 16S rRNA marker gene failed to reflect the multiplicity of these organisms in their sponge host and in freshwater. This outcome strengthens observations made for the soil environment on our biased perception of the diversity of pseudomonads in nature [33]. Through the parallel use of a cultivation-dependent approach, we substantiate this notion by examining the rarefied richness of *Pseudomonas* spp. isolated from *E. fluviatilis*. We found a much higher level of nucleotide diversification within the *gacA* in comparison with the 16S rRNA gene, leading to a more accurate, single gene-based coverage of full genome richness within the surveyed genotypes. Reversible *gacA* gene mutations and rearrangements underlie phenotypic variation within *Pseudomonas* spp., mediating their interaction with eukaryotic hosts [28]. Phase variation as a response to environmental stimuli might thus constitute an overriding force driving *Pseudomonas gacA* gene heterogeneities. Nevertheless, *gacA* gene tree topologies ([32], [33]; this study) mirror well the currently proposed phylogenetic relationships of the species in the genus [54], displaying thereby a higher resolving power in distinguishing closely related strains than the 16S rRNA gene.

*GacA* PCR-DGGE profiling further unveiled lowered diversity in *E. fluviatilis vs.* ambient water, resembling the overall trend for selective structuring of bacterial communities in this host [14]. Host-driven selection of bacteria is a well-known phenomenon in marine sponges [55]–[57] which awaits extensive verification in freshwater species [14]. We also depicted specimen-to-specimen and year-to-year variability in the structure of *Pseudomonas gacA* gene assemblages associated with *E. fluviatilis* individuals sampled in close proximity, along with the persistence of few phylotypes across all or several of these individuals. Previous molecular surveys of bacterial communities in freshwater sponges relied on snapshots of their structure/diversity from a single sampling event [14], [15], [58]. The consistent temporal and individual persistence of some pseudomonads in *E. fluviatilis* implies either the existence of a yet unknown mechanism of symbiont maintenance in these hosts or a highly efficient colonization capacity of specific opportunistic phylotypes. In contrast with marine species (the exception being the genus *Haliclona*), freshwater sponges in the families Metaniidae, Potamolepidae and Spongillidae (to which *E. fluviatilis* belongs) produce internal and asexual resting structures called gemmules [13] which allow the organism to withstand circumstances of environmental hardiness such as low temperatures, oxygen depletion and drought. In North- and central Europe, gemmules emmerge notably in autumn to resist unfavourable conditions, disperse and replicate. In spring, totipotent cells creep through foramina of an outer compact cuticle layer and generate the juvenile sponge [59]; see [13] for life cycle description at lower latitudes. Interestingly, *Spongilla lacustris* produces gemmules containing a few to large numbers of symbiotic algae [60], [61] which are beneficial to the developing sponge as they supply carbohydrates and oxygen [62]. Vertical symbiont transmission through sponge larvae is an important mechanism for the establishment of sponge-bacterial associations in several marine species [11], [56], [63]. However, it is so far not known whether bacterial symbionts are transmitted to the next freshwater sponge generation via gemmules and/or larvae, and questions emerge, *e.g.* if and how bacteria would be selected and which are the ‘sponge-specific’ bacteria to be transferred.

The second aim of this study was to assess the potential biotechnological value of *E. fluviatilis* as a novel source of pseudomonads with antimicrobial activity. We found a high frequency of *in vitro* antibacterial, antiprotozoan and antioomycetal activities among sponge-derived *Pseudomonas* isolates, and even some strains with the ability to suppress the growth of ravaging basidiomycetal and ascomycetal pests. Multivariate analysis discriminated highly antagonistic isolates in two groups, one more active against phytopathogenic fungi and *P. ultimum* and the other presenting toxicity against *B. subtilis* and *C. steinii*. This suggests in-faunal partitioning of antagonistic functions, allowing assumptions with respect to the bioactivity spectrum within sponge-inhabiting bacteria and their functional relevance. Metabolites from sponge symbionts attract considerable research interest due to their properties of potential pharmaceutical value [6]. Fluorescent *Pseudomonas* spp. produce several compounds of different classes showing strong antimicrobial activities such as glycolipids [64], cyclic lipopeptides [65], phenazines [66] and polyketides [37]. Antagonists tentatively identified in this study as *P. protegens*, *P. oryzihabitans* and *P. jessenii*, among others, matched dominant *gacA* gene fragments directly amplified from *E. fluviatilis* metagenomes. Antimicrobial features have been described for all these three species [67]–[69]. *P. protegens* strains Pf-5 and CHA0, the closest relatives of highly antagonistic strains of BOX-PCR cluster I, synthesize a range of inhibitory compounds such as pyrrolnitrin, 2,4-diacetyl-phloroglucinol, pyoluteorin and hydrogen cyanide [36], [37] which provide them with high competitive and anti-predatory capabilities in soil [25], [50], [70]. Notably, the genome of *P. protegens* Pf-5 is densely populated with secondary metabolite gene clusters [36], most of which with presumed horizontal gene transfer histories [37]. In this context, it is intriguing to note that the genomic structure underlying the biosynthesis of the antitumor polyketide onnamide A in the marine sponge *Theonella swinhoei* resembles that of a *Pseudomonas* symbiont of the terrestrial beetle *Padereus fuscipes* [7], [71], providing evidence for the maintenace of mobile genetic clusters involved in secondary metabolite biosynthesis across microbial symbiotic consortia. Recently, novel PKS encoding genes were reported for a *Pseudomonas* strain isolated from the freshwater sponge *Baikalospongia bacillifera* [35]. However, in spite of their documented co-dominance [72] and biocontrol capabilities [73], [74] in freshwater biomes, little is known about the genomic architecture and the spread and nature of secondary metabolite gene clusters of *Pseudomonas* species in these ecosystems. Collectively, our data suggest that the freshwater sponge *E. fluviatilis* is a promising source of underexplored *Pseudomonas* strains possessing a wide range of inhibitory activities whose underlying gene clusters and metabolites remain to be revealed.

Besides their biotechnological potential, a pertinent question concerns the ecological importance of inhibitory activities presented by a sponge-inhabiting pseudomonad. In addition, what could be the benefit of holding a heterogeneous, persistent and bioactive *Pseudomonas* community to the sponge host? In nature, antibiotics and other secondary metabolites serve multiple functions [75]. On the inhibitory route, toxic compounds impede bacteria to be phagocytised by amoebae [76], [77] and predated by ciliates, flagellates and/or nematodes [50], [70], [78]. They also enable bacteria to suppress the activity of competitors [26]. In addition, *in vitro* antagonistic compounds may in fact participate in bacterial cell-to-cell signalling and transcription modulation at realistic concentrations in the natural environment [79], [80]. The shape and dynamics of symbiont communities might therefore be largely affected by the cocktail of small molecules that are produced. Here, the ability of sponge-associated *Pseudomonas* spp. to produce biofilms in static microcosms was coincident with their antimicrobial potential (Fig. 7, Table S2). This is plausible since biofilm and antibiotic production are often regulated by the same mechanism and signalling molecules, *e.g.* homoserine lactones (HSLs). In plant-beneficial *Pseudomonas* spp., for instance, the expression of certain antibiotics depends on quorum sensing (QS) involving various HSLs [81], whereas in *P. aeruginosa* QS steered by HSLs is a premise for biofilm production [82]. A possible function of these products within the sponge environment could be driving off other microbes competing for habitat and nutrient availability. In addition, secondary metabolites might help pseudomonads to avoid being ingested via phagocytosis by archaeocytes, which are protist-like cells present in the host sponge responsible for nutrient uptake [49]. The capability of overcoming protozoan predation shown for several isolates in this study is indicative of a potential fitness-enhancing trait assisting the survivability of these microorganisms within the sponge, provided it does not compromise the viability of host cells. Alternatively, slime capsules, well known for the opportunistic pathogen *P. aeruginosa*, could also function as a structure enabling *Pseudomonas* spp. to evade engulfment by sponge cells and persist in this environment as previously suggested in a broader perspective [9], [62]. Tackling a possible benefit of hosting pseudomonads to the freshwater sponge host is a challenging task that deserves future research inspection. One possible scenario could be that the *Pseudomonas*-derived secondary metabolites might help the sponge to withstand attacks by bacterial or fungal pathogens or avoid microbial overgrowth, enhancing host fitness. Such bio-controlling or host-protecting roles have been widely described in *Pseudomonas*-plant symbiosis [25] and usually evoked as a likely function of secondary metabolites produced by marine sponge associated bacteria [11]. Although *Pseudomonas* spp. did not rank among the most common bacteria in *E. fluviatilis* as suggested by previous molecular analyses [14], their sharply enriched numbers in this organism, as revealed in this study, and status as a prevalent member of the culturable freshwater sponge microbiome [34] are indicative of a most likely active bacterium consortium inhabiting these hosts.

In summary, with a complementary cultivation-dependent and -independent approach, this study gives first insights into the ecology of pseudomonads in freshwater sponges. It suggests a distinct and mixed assemblage of both persistent and transient *Pseudomonas* spp. inhabiting the model organism *E. fluviatilis*. The increased abundance of these symbionts in the sponge host as compared with bulk water along with their wide genotypic and phenotypic heterogeneities highlight freshwater sponges as reservoirs of diverse *Pseudomonas* spp. with broad *in vitro* antimicrobial activity (Table S2) of potential biotechnological value, *e.g.* in the search for novel chemical structures with microbial inhibitory capacities. Future focus on the temporal stability of a wider range of microbial symbionts (e.g. the domains *Bacteria* and *Archaea*), and on their occurrence in/on resting structures such as gemmules, will shed further light on our understanding of the dynamics and evolution of the freshwater sponge holobiont. Further, ecogenomics of *Pseudomonas* species arises as a much needed approach to unveil their roles and adaptive strategies as host-associated and free-living bacteria in freshwater biomes. As genome sequencing of *Pseudomonas* spp. persists heavily biased towards animal and plant pathogens and soil-borne specimens, the sponge-associated strains reported here emerge as a promising source for novel bioactive secondary metabolites and future genome mining endeavours.

## Supporting Information

Table S1 **Closest 16S rRNA and** ***gacA*** **gene relatives of fluorescent** ***Pseudomonas*** **spp. isolated from** ***Ephydatia fluviatilis*** **.**
(XLSX)

Table S2 ***In vitro*** **antagonistic activity profiles of** ***Pseudomonas*** **spp. isolated from** ***Ephydatia fluviatilis*** **.**
(XLSX)
